# Is Novelty Detection Important in Long-Term Odor Memory?

**DOI:** 10.3390/brainsci11091146

**Published:** 2021-08-29

**Authors:** E. Leslie Cameron, E. P. Köster, Per Møller

**Affiliations:** 1 Department of Psychological Science, Carthage College, 2001 Alford Park Drive, Kenosha, WI 53140, USA; 2 Helmholtz Institute, University of Utretch, Wildforsterweg 4A, 3881NJ Putten, The Netherlands; ep.koster@gmail.com; 3 Per Møller Consulting, Langemosevej 17, 2880 Bagsvaerd, Denmark; p2moller@gmail.com

**Keywords:** memory, olfaction, vision, forced-choice methods

## Abstract

Memory for odors is believed to be longer-lasting than memory for visual stimuli, as is evidenced by flat forgetting curves. However, performance on memory tasks is typically weaker in olfaction than vision. Studies of odor memory that use forced-choice methods confound responses that are a result of a trace memory and responses that can be obtained through process of elimination. Moreover, odor memory is typically measured with common stimuli, which are more familiar and responses may be confounded by verbal memory, and measure memory in intentional learning conditions, which are ecologically questionable. Here we demonstrate the value of using tests of memory in which hit rate and correct rejection rate are evaluated separately (i.e., not using forced-choice methods) and uncommon stimuli are used. This study compared memory for common and uncommon odors and pictures that were learned either intentionally (Exp. 1) or incidentally (Exp. 2) and tested with either a forced-choice or a one-stimulus-at-a-time (“monadic”) recognition task after delays of 15 min, 48 h or 1 week. As expected, memory declined with delay in most conditions, but depended upon the particular measure of memory and was better for pictures than odors and for common than uncommon stimuli. For common odors, hit rates decreased with delay but correct rejection rates remained constant with delay. For common pictures, we found the opposite result, constant hit rates and decreased correct rejection rates. Our results support the ‘misfit theory of conscious olfactory perception’, which highlights the importance of the detection of novelty in olfactory memory and suggests that olfactory memory should be studied using more ecologically valid methods.

## 1. Introduction

Memory for odors has long been believed to be robust and long-lasting [1]. The longevity of olfactory memory is sometimes taken as evidence that it is special or unique compared to other forms of memory. This idea is supported by the fact that odors can evoke long-term autobiographical memories i.e., the Proustian phenomenon [2,3,4,5]. In *Remembrance of Things Past*, Marcel Proust described the smell of a madeleine (or, more accurately, its taste when dipped in linden tea) evoking a strong autobiographical childhood memory and most people are familiar with this experience. Interestingly, it has been reported that odors are more effective than visual cues at evoking autobiographical memories, e.g., [2,6] and that autobiographical memories evoked by odors (i.e., odor-evoked memories) go back to an earlier time in life than do memories evoked by visual stimuli [7,8]. The Proustian phenomenon has generated scientific interest and a recent review conducted by Hackländer et. al. [9] seems to demonstrate the olfactory system’s resistance to forgetting.

**Figure 1:**
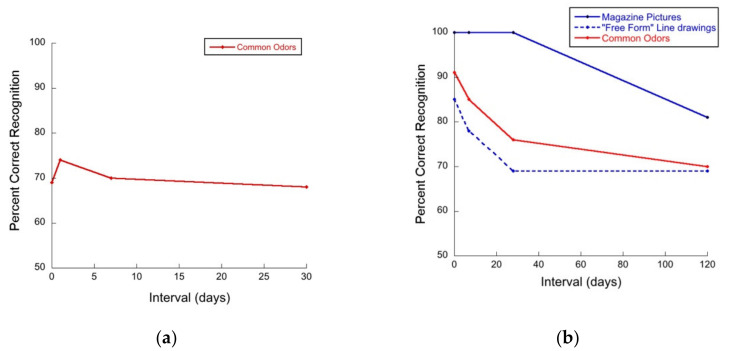
(**a**) Flat forgetting curve for common odors over 30 days, adapted from Engen and Ross [16]. (**b**) Initial decay of visual and odor recognition memory followed by flat forgetting curves for common odors and free form line drawings over time, adapted from Lawless [17].

Another form of long-term olfactory memory is odor recognition memory, e.g., [10,11,12,13]. This is memory for odors, per se. In tests of odor recognition memory [14,15], people are presented with one odor or a set of several odors and asked to recognize the odor or odors sometime later. Using such tasks, classic papers in the literature have reported forgetting curves for odors that are relatively “flat” [16,17,18,19]. For example, the results of Engen and Ross [16], as shown in Figure 1a, and those of Lawless [17], as shown in Figure 1b, demonstrate relatively little forgetting of odors over time. In the latter case, the flat forgetting curves follow an initial rapid decline for odors and free form pictures and a significant decline for pictures at longer delays. This has been taken as evidence for the longevity of odor memory.

Notwithstanding the flat forgetting curves, classic data in the odor memory literature do not support the notion that odor memory is better than visual memory in an absolute sense. For example, the data from Lawless [17] (see Figure 1b) demonstrated that the recognition of magazine pictures at short delays was perfect, while odors were recognized at a significantly lower rate, which was more similar to that of “free form” (FF) images. Likewise, more recently Cornell Kärnekull and colleagues [10] showed that memory for faces is better (higher d’ and hit rates) than memory for odors. Moreover, Cornell Kärnekull and colleagues [10] also reported forgetting curves for images and odors that were very similar (i.e., memory for both types of stimuli deteriorated similarly over time) consistent with Murphy and colleagues [20] and Olsson and colleagues [21], calling into question the widely held believe that memory for odors is long-lasting and immune to forgetting.

Odor recognition memory is likely be affected by a variety of parameters [22], such as the size of the set of odors tested [16], the similarity between targets and foils [1,23,24], and the hedonic valence of the odors [25]. The effect of odor familiarity and the ability to name odors on odor recognition memory is less clear. For example, Lawless and Cain [18] found little influence of these factors, whereas Rabin and Cain [19] and Stevenson and Mahmut [26] found that familiarity did affect odor recognition memory. Some studies have found that verbalization (i.e., the application of verbal labels to odors) may improve odor memory [19,24,26,27,28,29,30,31,32,33], but some have not [16,18,34]. Moreover, some researchers have argued that high level cognition is important for olfactory processing [35,36,37,38].

Not denying that top-down effects can be important in human olfactory processing, see [39], in the current study, we are interested in the critical question of whether there is a memory for the odor per se (veridical perceptual memory/template/trace) that bypasses odor naming, or if instead there is only a memory for odors that relies on the word that is associated with that odor (i.e., a semantic verbal memory). One way to avoid this possible confound experimentally is to use stimuli that are uncommon and very difficult to name [24,34,40,41]. Memory for such uncommon stimuli is unlikely to be confounded by verbalization.

An additional parameter that has received less attention in the literature on odor recognition memory involves the use of intentional and incidental learning paradigms. In many studies of odor recognition memory participants know that they are part of a memory study and can try to use focused encoding strategies to aid in their memory of stimuli. This is quite an artificial scenario compared to the way people typically learn about their sensory world, which is largely incidentally and thus the use of incidental learning paradigms are preferable in tests of odor memory [42].

In the current study, we re-examine odor and visual recognition memory over long delays (up to 1 week) with common and uncommon odors and pictures and under conditions of both incidental and intentional learning. This study was motivated by the question: Does odor memory rely on the same mechanisms as visual memory? Before addressing this question, we first outline a set of methodological and theoretical considerations.

### 1.1. Methodological Considerations

Tests of sensory memory, such as memory for odors or pictures, differ from tests of cognitive processes, such as verbal memory. It is difficult, if not impossible, to test recall of a sensory experience since people cannot conjure up and report a sensory experience in that modality, whereas it is possible to recall, and trivial to report the memory of a word, as such. One could argue that for visual and perhaps auditory stimuli (i.e., the “far senses”), memory can be tapped by the recall of very simple stimuli, such as by drawing a picture or reproducing a sound. This does not seem to be the case for the “near senses” (i.e., olfaction, taste or somatosensory perception). In this paper, we distinguish between “far” and “near” senses. For near senses the epithelium is in direct contact with the material properties of the stimulus (smell, taste, touch, etc.) whereas this is not the case for “far” senses (vision, audition). Recalling an odor may be particularly difficult because the ability to conjure up an “odor image” is less common in people than their ability to conjure up an image of a picture [43,44]. Thus, to test olfactory memory using recall, it would only be possible for people to remember and reproduce a label for an odor and not the odor per se. However, it is well known that verbal labeling of odors is overall quite poor and depends very much upon the particular stimuli [1,27,40,45,46]. Thus, tests of sensory memory typically employ recognition memory tasks, which may be impacted by verbal labeling, as noted above. This issue can be addressed by using uncommon odors that are difficult to name, as noted above.

In an *n*-alternative forced choice paradigm, a problem in interpretation of performance is that a correct response could indicate the recognition of the target odor (i.e., a hit in signal detection terms); however, it is also possible that the target odor was selected because the participant recognized that the other odors, hereafter referred to as foils, were not presented previously, see [19]. In other words, a correct response could reflect a process of elimination—novel odors (foils) were detected and rejected. This is important because selecting an odor as being remembered can be interpreted as some sort of template or trace memory, whereas selecting an odor because foils were not recognized would reflect a different mechanism, namely the detection of something new or novel.

There are, at least, two ways to address this issue methodologically. First, one could give participants the option of saying that “neither” of the presented stimuli were the target in a 2-AFC task. A “neither” response stops a participant from being forced to make a false positive. A false positive would be a response made when the participant has detected that the foil is a novel odor, and thus selects the other odor as the target. A second alternative method of testing odor memory is a so-called “monadic” version of the test phase, which is effectively a “yes/no” task, see [24]. In such a task, odors are presented one at a time and the participant responds whether that odor is one they smelled in the test phase (“yes”) or not (“no”). A benefit of using a monadic task compared to a forced-choice task, is that signal detection analyses can be conducted. The separate examination of sensitivity, bias, hit rates and correct rejection rates provides additional information about the processes or mechanisms by which memory operates. For example, hit rates in the absence of bias presumably reflect a memory template or trace for a stimulus, whereas correct rejection rates are an indication that a stimulus has been recognized as new.

### 1.2. Theoretical Considerations

The mechanisms involved in olfactory memory may be different from those in other memory systems [47]. According to these authors’ theory, and in agreement with the mnemonic odor memory theory of Stevenson and Boakes [48], odors are immediately, but unconsciously, qualified as new or as ‘has been perceived before’ as an a priori step in the safeguarding function of olfaction. Olfaction protects our most vital functions, including breathing, food uptake, avoidance of poisoning [49]. Furthermore, Köster and colleagues [47] argue that, in order to fulfill that function, odors are immediately strongly associated with the situation in which they occur. As a result, upon reappearance of an odor, the previous situation is also recalled (i.e., the Proustian phenomenon, as described above) and inversely if the situation repeats itself, deviations in the odor immediately draw our attention, whereas the original odor in that situation usually goes unnoticed. Thus, the theory is that novelty or change detection, rather than odor template memory is the predominant mechanism of olfactory memory. This is in contrast to visual memory where recognition of the template prevails. ‘Novelty’ detection still requires some trace/template of odors from previous exposures. However, these templates need not be represented at a ‘higher level’ of the olfactory system, but could reside at the level of the olfactory bulb as suggested by Stevenson and Boakes [48]. As noted above, these authors suggest that odors are distinguished at this preconscious level as ‘novel’ or not novel. This is different from memory traces of previously perceived odors which are also represented at higher levels of the system.

In this paper we address the question: Does olfactory memory rely on different mechanisms than visual memory? In particular, we ask whether “novelty detection” is an important mechanism in long-term olfactory memory? We address this by examining memory for common and uncommon pictures and odors for up to one week in both intentional and incidental learning conditions, by giving participants the option of a “neither” response in a forced-choice task and by examining performance in a “monadic” test paradigm. We discuss the results of our experiments in the context of the “Misfit Theory of Spontaneous Conscious Odor Perception” MITSCOP, [47], which suggests that novelty detection is an important mechanism in olfactory memory.

The research hypotheses were:

1. Visual and olfactory memory depend differentially on ‘novelty’ detection as will be demonstrated with differential hit and correct rejection rates and patterns of responses with delay in the monadic “yes/no” task.
2. Olfactory memory suffers less forgetting than visual memory. We expect to find main effects of delay for pictures but not for odors.
3. Memory of uncommon stimuli decays similarly to memory of common stimuli, as demonstrated with no interactions between the variables of delay and “commonness”.

## 2. Materials and Methods (Intentional Learning Condition)

This condition was designed to ask whether novelty detection is important in olfactory memory by using a two-alternative forced choice task with the option of a “neither” response and by examining performance in a “monadic” test paradigm. Memory was tested within a typical intentional learning paradigm over delays between 15 min and 1 week.

### 2.1. Participants

One hundred and sixty-five young adults (49 males (20.88 years) and 116 females (19.55 years) all between 18 and 23 years of age) participated in this experiment and each participated in only one delay condition. Most participants were non-smokers (the exceptions were—1 male smoker and 2 males who smoke occasionally; 11 females had smoked in the past, and 1 was an occasional smoker). Participants were recruited from individual classes (with instructor permission), via email, through word of mouth, and via an online college bulletin board. Participants were compensated with a $10 gift card upon completion of the memory task.

### 2.2. Materials

Appendix ATable A1 lists the odor stimuli, consisting of common household items (e.g., chocolate) and uncommon chemical compounds (e.g., Citrowanil). Common odors were chosen from a set used in previous odor memory and odor identification studies [40,45,50], spanned a range of discriminability and were purchased from a local grocery store. The uncommon odors have been used in previous research [34,40,41] and were obtained from International Flavors and Fragrances (IFF). Odors were presented at roughly the same suprathreshold intensity (determined in pilot testing). The odors were presented in 30 mL amber-colored jars with a numerical code on the lid. Each jar was also covered in aluminum foil to further conceal the contents. Participants were instructed not to look in the jars.

**Figure A1:**
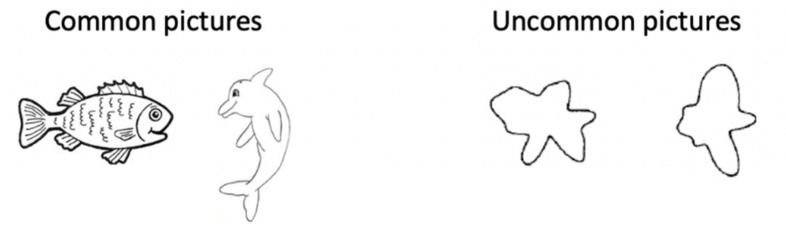
Sample pictures.

The common pictures (e.g., shapes, animals and furniture) and the uncommon, free-form pictures (see Appendix A Figure A1) taken from Bromley and Doty (15) and Lawless [17] were printed on the inside of white folded cardstock. Pictures were between 1 × 1 to 1.5 × 1.5 inches in size. Target and foils were paired by their commonness and similarity and were the same odors employed previously in our lab [40].

### 2.3. Procedure

Participants were tested in small groups (1–7 people) in a well-ventilated classroom or lab setting. The experimenter told the participants that this was a memory test and that they should make every effort to remember the stimuli. The memory test occurred after a delay of 15-min, 48-h, or 1-week during which time there was no control over what participants did.

In Phase I, participants were presented with a set of 20 odors and 20 pictures (odors and pictures were interleaved to reduce the likelihood of adaptation and the order of common and uncommon stimuli was counterbalanced) and rated the familiarity of each stimulus, using a Likert scale (1–9). Participants opened each jar, one at a time, sniffed the contents, privately wrote a familiarity rating, recapped the jar and handed it to the person to their right. They then opened a card and viewed a picture, rated the familiarity, closed the card and passed it to the right. This continued until all participants had smelled and rated each jar and each picture. The experimenter controlled the speed of testing, which was about one jar or picture every 10–15 s, thus there were about 30 s between odor exposure. Stimulus presentation order was not identical for each person. Familiarity ratings were used to encourage participants to attend to the stimuli in the learning phase of this experiment, and confirmed that common stimuli were rated as more familiar than uncommon ones. They confirmed that common and uncommon stimuli were rated as such. Mean familiarity ratings were as follows: common odors (6.15), uncommon odors (4.34), common pictures (8.43) and uncommon pictures (3.85).

In Phase II (a recognition memory test), participants reconvened in the same group after either 15 min, 48 h or 1 week, in the same room and were presented with 40 odors and 40 pictures. Odor memory was tested using one of two methods: (1) two-alternative forced-choice task with option of a “neither” response: Participants received stimuli in pairs (sequentially) and indicated which odor or picture in the pair had been presented in Phase I. The experimenter kept the trials going at a swift pace, so participants were limited in the amount of time they could sample the stimuli. “Neither” was a response option, which participants could use to indicate that they did not think either stimuli had been presented previously. Unbeknownst to the participants, “neither” was never the correct response as one of the stimuli had always been presented. Pairs of odors and pictures were either common or uncommon and never mixed across category. The order of pairs of stimuli was randomized so that the target was presented equally often as the first or second stimulus. (2) Monadic task: Participants received stimuli one at a time and their task was to indicate whether or not the stimulus had been presented in Phase 1. This was a between-subjects design, so participants either participated in a 2-AFC or monadic task, not both.

This experiment received approval from the Carthage College Institutional Review Board and was conducted in accordance with the ethical principles regarding human experimentation, as outlined in the Declaration of Helsinki. All participants gave written informed consent.

## 3. Results (Intentional Learning Condition)

### 3.1. Forced Choice Task

**Figure 2:**
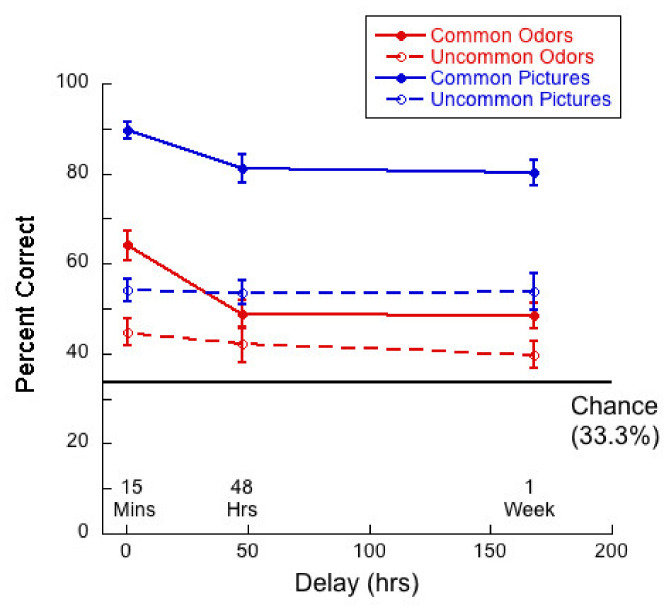
Percent correct as a function of delay for each of the four test conditions. Chance performance is 33.33% because participants could make a “neither” response. Here and in all figures, error bars reflect ± standard error of the mean. There were 27 participants in the 15-min delay condition, 25 at 48 h and 27 at 1 week.

Figure 2 shows percent correct as a function of delay for the four stimulus types when stimuli were presented in an intentional learning condition.

We had no predictions about the difference in absolute level of performance of pictures and odors (these could have been made more or less similar by manipulating the stimuli), thus we ran separate analyses for pictures and odors for this and all analyses.

A 2 (commonness) × 3 (delay) mixed ANOVA on percent correct for the picture stimuli revealed no significant interaction (*F*(2,76) = 1.43; *p =* 0.25, η^2^ = 0.04) between commonness and delay. There was a main effect of commonness (*F*(1,76) = 155.63, *p* < 0.001, η^2^ = 0.67), but not of delay (*F*(2,76) = 1.73, *p* = 0.19, η^2^ = 0.04). Thus, performance was much higher for common than for uncommon pictures, but performance did not decline significantly with delay.

A 2 (commonness) × 3 (delay) mixed ANOVA of percent correct for the odor stimuli also revealed no significant interaction (*F*(2,76) = 2.23; *p =* 0.11, η^2^ = 0.06) between commonness and delay. However, there were main effects of both commonness (*F*(1,76) = 20.13, *p* < 0.01, η^2^ = 0.21), and delay (*F*(2,76) = 6.18, *p* < 0.01, η^2^ = 0.14). Thus, performance was much higher for common than for uncommon odors and performance declined with delay. Tukey post hocs for multiple comparisons indicated significant differences between the 15-min and 48-h condition (*p =* 0.02) and between the 15-min and 1-week (*p <* 0.01), but not for the difference between 48 h and 1-week (*p* = 0.92).

The forced-choice method used in the current study, in which the participant could respond that “neither” of the test stimuli were the target, means that there are two possible ways in which a participant could have made an error: (1) They could have chosen the foil, which could be interpreted as meaning that any potential template does not have high fidelity, or (2) they could have chosen to report that neither of the odors in the test phase was the target. In this case, the foil was correctly evaluated as novel and the target was, incorrectly, evaluated as novel.

We ask two questions about the “neither” responses in the current data. First, we explored how many “neither” responses were made overall for each stimulus type. For common and uncommon pictures, the rates of “neither” responses were 12.81% and 21.26%, respectively. For common and uncommon odors, the rates of “neither” responses were 32.27% and 13.92%, respectively. Only ~6% of participants never used the option of “neither”. Without the option “neither”, such as is in a typical 2AFC task, contributions to apparently correct target detections can arise in the following ways: (1) By relying on a memory trace of the target (a true ‘recognition’ of the target); (2) by detecting that the foil is a novel target and thereby deduce that the other test stimulus is the target; and (3) by guessing correctly in cases where both stimuli appear to be ‘novel’, i.e., that neither of the stimuli are recognized as having been smelled before, participants will have to make a random choice, thereby inflating apparent correct target detection with half of these guesses. For common odors in the present experiment this contribution was about 16% (averaged over delay). The percent correct responses for common odors shown in Figure 2 would therefore be about 16% higher in a normal 2AFC experiment without the response option ‘neither’.

**Figure 3:**
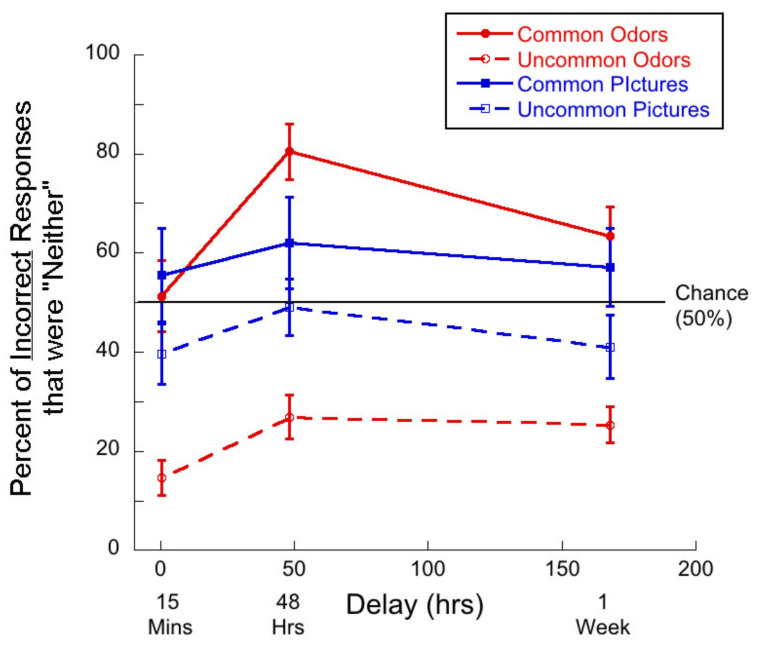
Percentage of incorrect responses that were “neither” responses as a function of delay for each of the four test conditions. Note that “neither” was never a correct response. Given that there were two ways to arrive at an error, chance is 50%.

Second, we ask whether “neither” responses make up more than half of all incorrect responses. Such responses indicate that participants perceived both the target odor and the foil as novel. Figure 3 shows the percent of incorrect responses that were “neither” as a function of delay for the four stimulus types.

In the case of common odors, single-sample t-tests demonstrated that at the 48-h and 1-week delays, participants were more likely to choose “neither” than chance (50%). The mean at 48 h was 80.45% (*t*(24) = 5.39, *p <* 0.01, *d* = 1.08) and the mean at 1 week was 63.35% (*t*(26) = 2.24, *p =* 0.02, *d* = 0.43).

In the case of uncommon odors, the rates of “neither” responses were significantly lower than chance. At 15 min, 14.67% < 50% (*t*(26) = 10.04, *p <* 0.01, *d =* 1.93) at 48 h 26.88% < 50% (*t*(24) = 5.21, *p <* 0.01, *d =* 1.04) and at 1 week, 25.35% < 50% (*t*(26) = 6.77, *p <* 0.01, *d =* 1.32)

For common pictures, the percent of incorrect responses that were “neither” were not significantly different from chance in any delay condition.

### 3.2. Monadic Task

**Figure 4:**
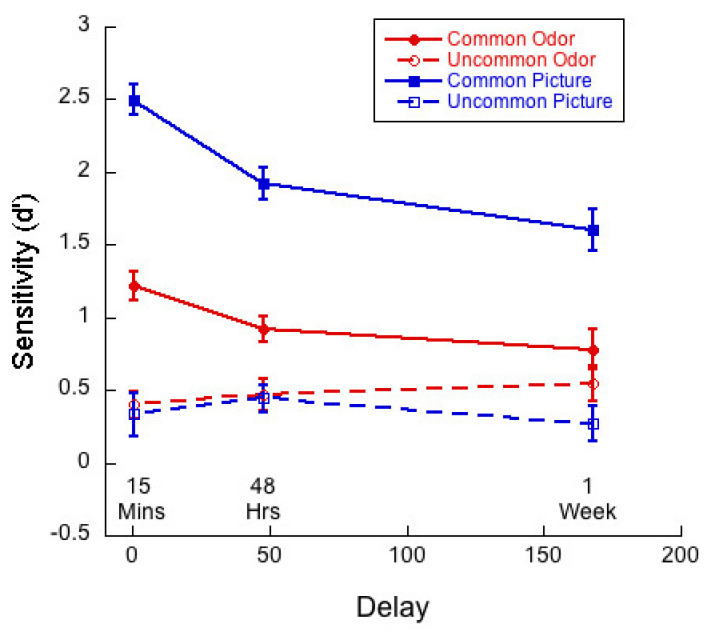
Sensitivity/d-prime (d’) as a function of delay for each of the four test conditions. There were 29 participants in the 15-min condition, 30 at 48 h and 27 at 1 week.

Figure 4 shows sensitivity/d-prime (d’) as a function of delay for common and uncommon pictures and odors. (d’ values were calculated using the formula: d’ = z (hit rate)–z (false alarm rate). Given that hit and false alarm rates of 1 and 0 cannot be used in this equation, calculation rates of 0.9444 and 0.0555 were substituted for rates of 1 and 0, respectively, following Stanislaw and Todorov [51]).

A 2 (commonness) × 3 (delay) mixed ANOVA of d’ for the picture stimuli revealed a significant interaction (*F*(2,83) = 2.83; *p <* 0.01, η^2^ = 0.17). There was a main effect of commonness (*F*(1,83) = 341.48, *p* < 0.01, η^2^ = 0.80), and of delay (*F*(2,83) = 6.41, *p* < 0.01, η^2^ = 0.13). Univariate analyses conducted for the common and uncommon pictures separately indicated that the effect of delay was significant for the common stimuli only (*F* (2, 83) = 14.2, *p <* 0.01, η^2^ = 0.26). Tukey post hocs for multiple comparisons indicated that the difference between the 15-min condition and the 48-h (*p <* 0.01) and 1-week (*p <* 0.01) conditions were significant, but the difference between 48 h and 1-week conditions were not (*p* = 0.15).

A 2 (commonness) × 3 (delay) mixed ANOVA of d’ for the odor stimuli revealed a significant interaction (*F* (2, 83) = 4.21; *p <* 0.02, η^2^ = 0.09). There was a main effect of commonness (*F*(1,83) = 36.89, *p <* 0.01, η^2^ = 0.31), but the effect of delay was not significant (*F* (2, 83) = 0.86, *p =* 0.43, η^2^ = 0.02). Univariate analyses conducted for the common and uncommon odors separately indicated that the effect of delay was significant for the common stimuli only (*F*(2,83) = 4.02, *p =* 0.02, η^2^ = 0.09). Tukey post hocs for multiple comparisons indicated that the only significant difference was between the 15-min and 1-week conditions (*p =* 0.019).

**Figure 5:**
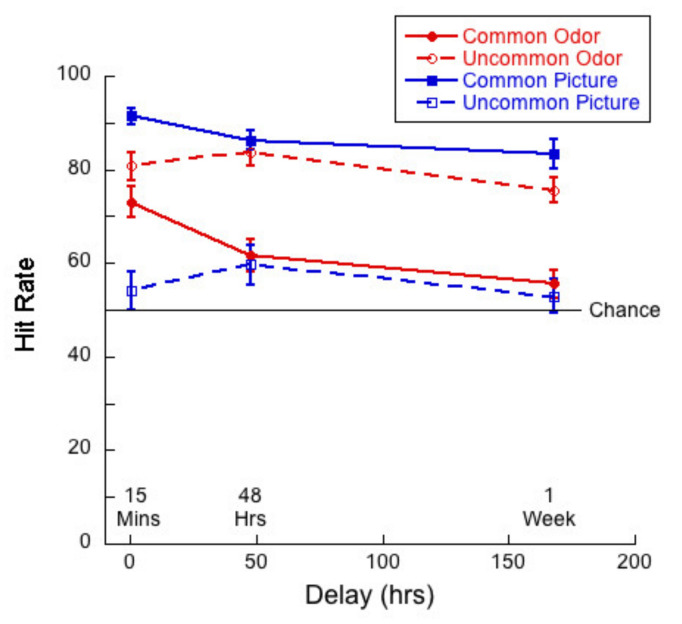
Hit rate as a function of delay for each of the four test conditions.

We analyzed the effect of delay on hit rates and correct rejections to explore the extent to which people had some sort of trace or template memory (hit) and the extent to which they are relying on novelty detection (correct rejection). Figure 5 shows hit rate as a function of delay for common and uncommon pictures and odors.

A 2 (commonness) × 3 (delay) mixed ANOVA of hit rates for the picture stimuli revealed no significant interaction (*F*(2,83) = 1.61; *p =* 0.21, η^2^ = 0.04). There was a significant main effect of commonness (*F*(1,83) = 161.59, *p <* 0.01, η^2^ = 0.66), but not of delay (*F*(2,83) = 1.09, *p =* 0.34, η^2^ = 0.03). Thus, hit rates were significantly higher for common than uncommon pictures, but did not decline significantly over one week.

By contrast, a 2 (commonness) × 3 (delay) mixed ANOVA of hit rates for the odor stimuli revealed a significant interaction (*F*(2,83) = 3.30; *p =* 0.04, η^2^ = 0.07) between commonness and delay. The main effects were both significant—delay (*F*(2,83) = 6.90, *p <* 0.01, η^2^ = 0.14) and commonness (*F*(1,83) = 43.6, *p <* 0.01, η^2^ = 0.35). Univariate analyses conducted for the common and uncommon odors separately indicated that the effect of delay was significant for the common stimuli only (*F*(2,83) = 7.18, *p <* 0.01, η^2^ = 0.15). Tukey post hocs for multiple comparisons indicated significant differences between the 15-min and 48-h condition (*p =* 0.04) and between the 15-min and 1-week (*p <* 0.01), but not for the difference between 48 h and 1-week (*p* = 0.40). The higher hit rates for uncommon odors are reflected in their bias scores (see below).

Although we recognize the limitations of direct comparisons between performance on our odor and picture tasks, in order to address the question as to the difference between visual and olfactory memory, have selected a few cases where we thought comparisons were meaningful. For example, we conducted a 2 (stimulus type) × 3 (delay) mixed ANOVA of the hit rates for common pictures and odors. In the case of hit rates, we found no interaction between our variables (*F*(2,83) = 1.38, *p* = 0.26, η^2^ = 0.03). There was a main effect of stimulus type (*F*(1,83) = 99.4, *p* < 0.01, η^2^ = 0.55) as memory was better for pictures than for odors, and a main effect of delay (*F*(2,83) = 9.93, *p* < 0.01, η^2^ = 0.19). Tukey post hocs for multiple comparisons indicated significant differences between the 15-min and 48-h condition (*p =* 0.01) and between the 15-min and 1-week (*p <* 0.01), but not for the difference between 48 h and 1-week (*p* = 0.26).

**Figure 6:**
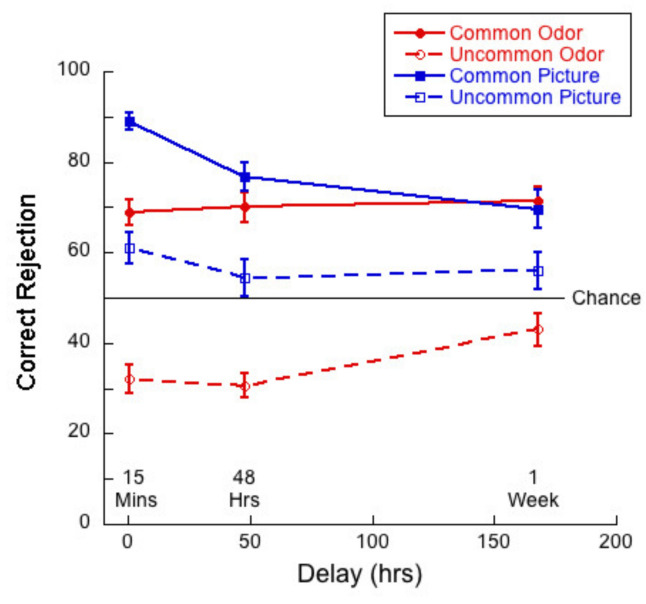
Correct rejection rate as a function of delay for each of the four test conditions.

Figure 6 shows correct rejection (CR) rates as a function of delay for common and uncommon pictures and odors.

A 2 (commonness) × 3 (delay) mixed ANOVA of CR rates for the picture stimuli revealed no significant interaction (*F*(2,83) = 2.64; *p =* 0.08, η^2^ = 0.06). There was a main of commonness (*F*(1,83) = 71.90, *p <* 0.01, η^2^ = 0.46), and of delay (*F*(2,83) = 5.07, *p <* 0.01, η^2^ = 0.11). Thus, correct rejection rates were significantly higher for common than for uncommon pictures and *decreased* significantly after one week. Tukey post hocs for multiple comparisons indicated a significant difference between the 15-min and 1-week delay conditions (*p =* 0.01) and a narrowly significant difference between the 15-min and 48-h conditions (*p* = 0.05), but no significant difference between the 48 h and 1-week conditions (*p* = 0.78).

A 2 (commonness) × 3 (delay) mixed ANOVA of CR rates for the odor stimuli revealed no significant interaction (*F*(2,83) = 2.07; *p =* 0.13, η^2^ = 0.05) between commonness and delay. There was a main effect of commonness (*F*(1,83) = 240.3, *p <* 0.01, η^2^ = 0.74), and a trend towards an effect of delay (*F*(2,83) = 2.53, *p =* 0.09, η^2^ = 0.06). Thus, CR rates were significantly higher for common than for uncommon odors and did not decrease with delay.

Again, to address the question as to the difference between visual and olfactory memory, we conducted a 2 (stimulus type) × 3 (delay) mixed ANOVA of the CR rates for common pictures and odors. In the case of CR rates, we found a significant interaction between variables (*F*(2,83) = 7.25, *p* < 0.01 η^2^ = 0.15). There was a main effect of stimulus type (*F*(1,83) = 12.48, *p* < 0.01, η^2^ = 0.13) as memory was better for pictures than for odors, and a marginally significant main effect of delay (*F*(2,83) = 3.15, *p* = 0.05, η^2^ = 0.07). Univariate analyses conducted for picture and odor conditions separately indicated that the effect of delay was significant for the pictures only (*F*(2,83) = 9.25, *p <* 0.01, η^2^ = 0.18). Tukey post hocs for multiple comparisons indicated significant differences between the 15-min and 48-h condition (*p =* 0.02) and between the 15-min and 1-week (*p <* 0.01), but not for the difference between 48 h and 1-week (*p* = 0.27). Thus, the pattern of memory performance was that CR rates decreased as a function of delay for pictures but not for odors.

**Figure 7:**
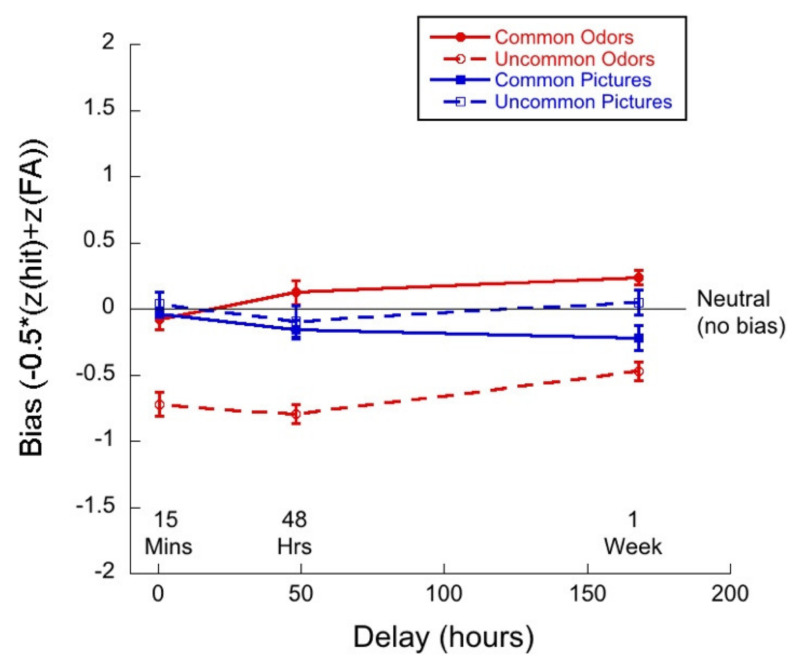
Bias as a function of delay for each of the four test conditions.

Figure 7 shows bias or criterion as a function of delay for common and uncommon pictures and odors. We calculated bias/criterion (c) using the following equation: −0.5 ∗ (Z(Hit) + z(FA), see [51,52].

A 2 (commonness) × 3 (delay) mixed ANOVA of the criterion for the picture stimuli revealed no significant interaction (*F*(2,83) = 1.06; *p =* 0.35, η^2^ = 0.03). There was a main effect of commonness (*F*(1,83) = 4.65, *p =* 0.03, η^2^ = 0.05), but not of delay (*F*(2,83) = 0.88; *p =* 0.42, η^2^ = 0.02).

A 2 (commonness) × 3 (delay) mixed ANOVA of the criterion for the odor stimuli revealed no interaction (*F*(2,83) = 1.99; *p =* 0.14, η^2^ = 0.05). There was a main effect of commonness (*F*(1,83) = 162.71, *p <* 0.01, η^2^ = 0.66), and a significant effect of delay (*F*(2,83) = 6.16, *p <* 0.01, η^2^ = 0.13).

Taken together, the most noteworthy result of the bias analyses is that the response criterion was very liberal for uncommon odors. Participants produced high hit rates (in fact, even higher than for common odors) and also produce remarkably low correct rejection rates. With delay, correct rejection rates for odors appears to remain constant or rise slightly.

## 4. Materials and Methods (Incidental Learning Condition)

### 4.1. Participants

One hundred and sixteen adults volunteered for this study. One hundred and fifteen completed the study—46 males (21.33 years) and 70 females (19.82 years), mostly between 18 and 22 years of age. There were three older females (29, 32 and 34 years of age) and one 54-year-old male participant). Participants were recruited via an email listserv and an online bulletin service of the college at three separate times. Data from one participant with self-reported anosmia were excluded from analysis. Most participants were non-smokers (the exceptions were—1 female smoker, 2 females and 3 males who smoke occasionally; 4 females and 4 males who had smoked occasionally in the past). Those with interfering allergy or cold symptoms were rescheduled for a later date. Participants were instructed not to consume anything but water an hour before the testing session. Small gifts (value < $5) were given as compensation.

### 4.2. Materials and Procedure

Testing was conducted as in the intentional learning condition except that participants were not told that they were in a memory experiment, but rather that there was a second phase of the experiment without specifying what the task would involve. Phase II was the same recognition memory test and participants were either tested with the forced-choice or monadic version of the task after a delay of either 15 min, 48 h or 1 week.

## 5. Results (Incidental Learning Condition)

### 5.1. Forced-Choice Task

**Figure 8:**
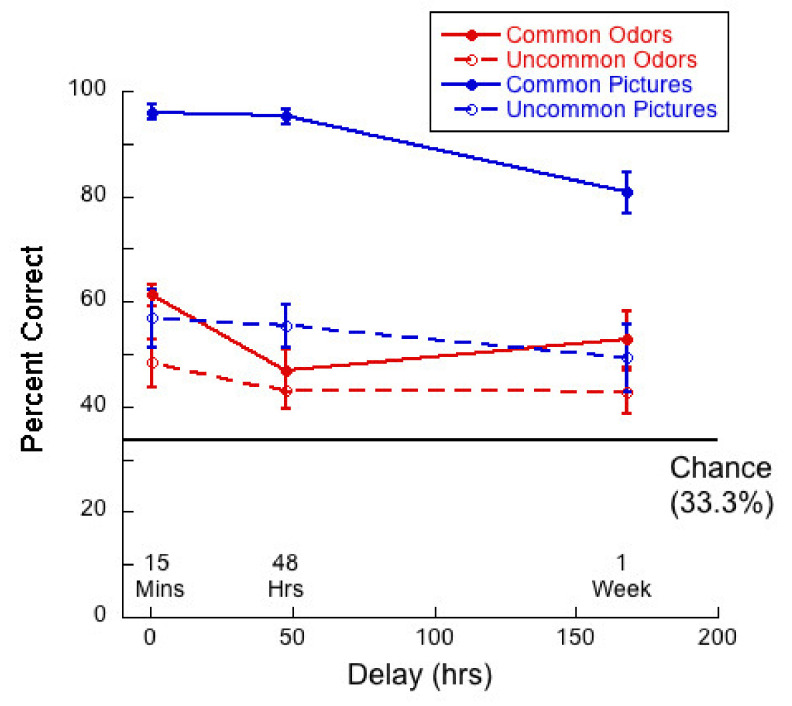
Percent correct as a function of delay for each of the four test conditions. Because there were three possible responses (“Odor 1”, “Odor 2” or “neither”), the chance was 33.33%. There were 22 participants in the 15-min delay condition, 19 at 48 h and 14 at 1 week.

Figure 8 shows percent correct as a function of delay for common and uncommon pictures and odors.

A 2 (commonness) × 3 (delay) mixed ANOVA on percent correct for the picture stimuli revealed no significant interaction (*F*(2,52) = 0.62; *p* = 0.54, η^2^ = 0.02) between commonness and delay. There was a main effect of commonness (*F*(1,52) = 125.98, *p* < 0.01, η^2^ = 0.71), and of delay (*F*(2,52) = 3.84, *p* = 0.03, η^2^ = 0.13). Thus, performance was significantly higher for common than for uncommon pictures and performance declined with delay. Tukey post hocs for multiple comparisons indicated no significant difference between the 15-min and 48-h condition (*p =* 0.96), nor between 48 h and 1-week (*p* = 0.07) but there were significant differences between the 15-min and 1-week (*p* = 0.03) delay conditions.

A 2 (commonness) × 3 (delay) mixed ANOVA on percent correct for the odor stimuli revealed no significant interaction (*F*(2,52) = 1.31, *p* = 0.33, η^2^ = 0.04) between commonness and delay. There was a main effect of commonness (*F*(1,52) = 10.61, *p* < 0.01, η^2^ = 0.17), and a trend for delay (*F*(2,52) = 2.80, *p* = 0.07, η^2^ = 0.10). Thus, performance was higher for common than for uncommon odors, but performance did not decline significantly with delay.

As we did for the intentional learning condition, we explored how many “neither” responses were made *overall* for each stimulus type. For common and uncommon pictures, the rates of “neither” responses were 9.67% and 27.72%, respectively. For common and uncommon odors, the rates of “neither” responses were 26.18% and 15.07%, respectively.

**Figure 9:**
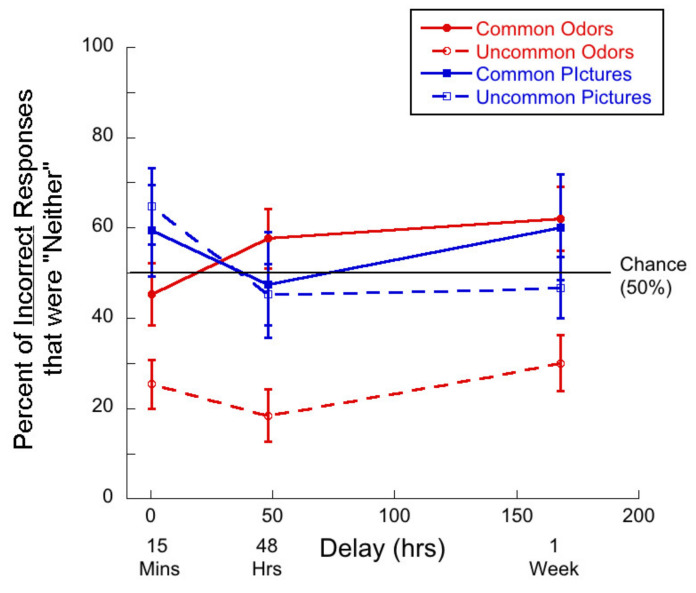
Percentage of “neither” responses as a function of delay for each of the four test conditions. Note that “neither” was never a correct response.

Figure 9 shows the percent of incorrect responses that were “neither” as a function of delay for the four stimulus types.

The pattern of “neither” responses is similar to the results of the intentional learning condition, but there was only a trend for the “neither” responses for common odors to be greater than chance at the longer delays. Only ~3.5% of participants never used the option of “neither”. For common odors, single-sample t-tests demonstrated that at the 48-h and 1-week delays, there was a trend towards participants choosing the “neither” response more often than chance. At 48 h, the mean percent of “neither” responses was 57.62% (*t*(18) = 1.15, *p* = 0.13, *d* = 0.26) and at 1 week, it was 62.02% (*t*(13) = 1.70, *p* = 0.06, *d =* 0.45).

In the case of uncommon odors, the relatively low rates of “neither” responses were significantly lower than chance. At 15 min, 25.41% < 50% (*t*(21) = 4.55, *p* < 0.01, *d* = 0.97), at 48 h 18.38% < 50% (*t*(19) = 5.43, *p* < 0.01, *d =* 1.25) and at 1 week, 30.01% < 50% (*t*(13) = 3.22, *p* < 0.01, *d* = 0.86).

For common pictures, the percent of incorrect responses that were “neither” were not significantly different from chance in any delay condition.

### 5.2. Monadic Task

**Figure 10:**
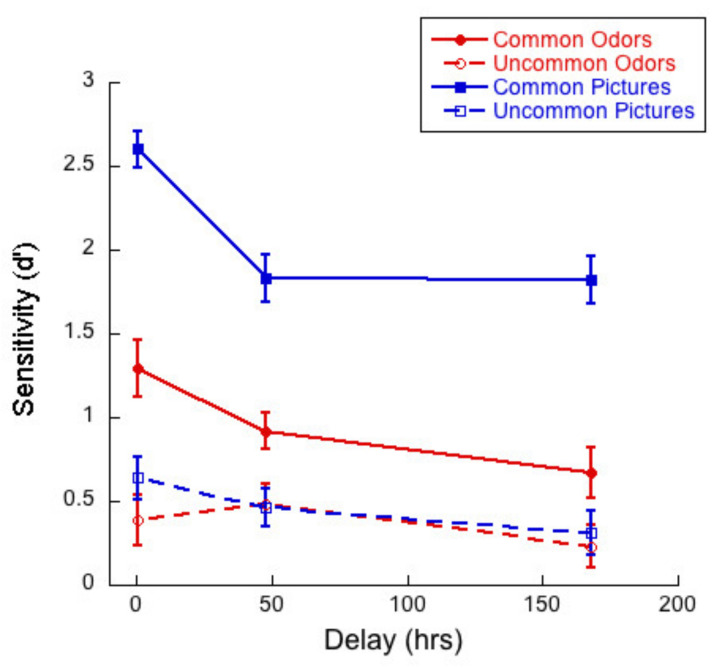
Sensitivity (d’) as a function of delay for each of the four test conditions. There were 20 participants in each delay condition.

Figure 10 illustrates sensitivity (d’) as a function of delay for common and uncommon pictures and odors.

A 2 (commonness) × 3 (delay) mixed ANOVA on d’ for the picture stimuli revealed a significant interaction (*F*(2,57) = 3.74; *p* = 0.03, η^2^ = 0.12) between commonness and delay. There was a main effect of commonness (*F*(1,57) = 300.0, *p* < 0.01, η^2^ = 0.84), indicating that d’ was higher for common than for uncommon pictures. There was also a main effect of delay (*F*(2,57) = 9.79, *p* < 0.01, η^2^ = 0.26). Univariate analyses conducted for the common and uncommon pictures separately indicated that the effect of delay was significant for the common stimuli only (*F*(2,57) = 4.04, *p <* 0.01, η^2^ = 0.30). Tukey post hocs for multiple comparisons indicated significant differences between the 15-min and 48-h condition (*p <* 0.01) and between the 15-min and 1-week (*p <* 0.01) but not for the difference between 48 h and 1-week (*p =* 1.0).

A 2 (commonness) × 3 (delay) mixed ANOVA on d’ for the odor stimuli revealed no significant interaction (*F*(2,57) = 2.26; *p* = 0.11, η^2^ = 0.07) between commonness and delay. There was a main effect of commonness (*F*(1,57) = 34.37, *p* < 0.01, η^2^ = 0.38) and of delay (*F*(2,57) = 3.50, *p* = 0.04, η^2^ = 0.11). Thus, sensitivity was higher for common than for uncommon odors and sensitivity decreased with delay. Tukey post hocs for multiple comparisons indicated significant differences only between the 15-min and 1-week condition (*p* = 0.03)

**Figure 11:**
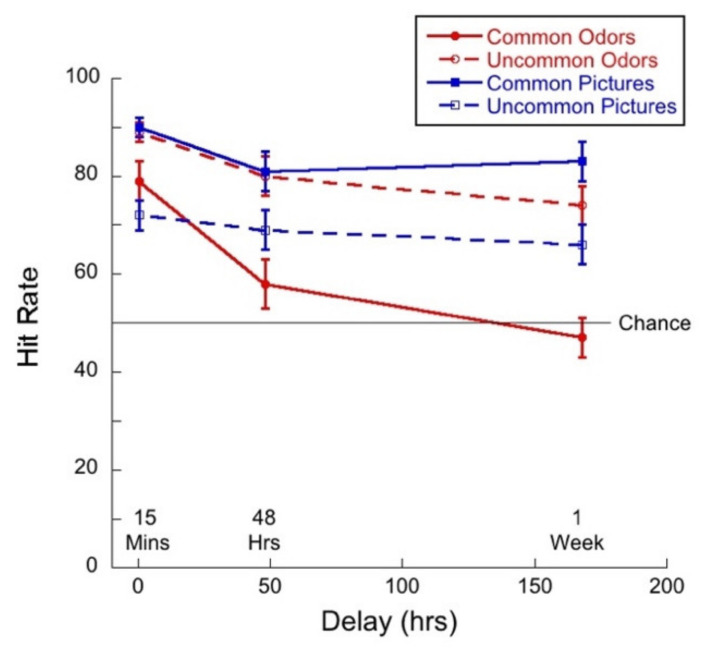
Hit rate as a function of delay for each of the four test conditions. Given that this was a “monadic” task, guessing would result in a hit rate of 50%.

Figure 11 shows hit rate as a function of delay for common and uncommon pictures and odors.

A 2 (commonness) × 3 (delay) mixed ANOVA on hit rate for the picture stimuli revealed no significant interaction (*F*(2,57) = 0.35, *p* = 0.71, η^2^ = 0.01) between commonness and delay. There was a main effect of commonness (*F*(1,57) = 28.12, *p* < 0.01, η^2^ = 0.33), and a trend towards an effect of delay (*F*(2,57) = 2.69, *p* = 0.08, η^2^ = 0.09). Thus, performance was higher for common than for uncommon pictures and performance declined, but not significantly, with delay.

A 2 (commonness) × 3 (delay) mixed ANOVA on hit rate for the odor stimuli revealed no significant interaction (*F*(2,57) = 2.15; *p* = 0.13, η^2^ = 0.07) between commonness and delay. There was a main effect of commonness (*F*(1,57) = 35.98, *p* < 0.01, η^2^ = 0.38), and a main effect of delay (*F*(2,57) = 21.9, *p* < 0.01, η^2^ = 0.44). Thus, hit rate was higher for uncommon than for common odors and hit rate declined significantly with delay for both conditions. Tukey post hocs for multiple comparisons indicated a significant difference between the 15-min and 48-h condition (*p* < 0.01), and between the 15-min and 1-week (*p* < 0.01), but not between 48 h and 1-week (*p* = 0.06).

It is important to note that hit rates for common stimuli dropped to chance after one week and that the hit rate for uncommon odors was very high.

We conducted a 2 (stimulus type) × 3 (delay) mixed ANOVA of the hit rates for common pictures and odors. We found a significant interaction between variables (*F*(2,57) = 6.85, *p* < 0.01 η^2^ = 0.19). There was a main effect of stimulus type (*F*(1,57) = 77.68, *p <* 0.01, η^2^ = 0.57) as memory was better for pictures than for odors, and a significant main effect of delay (*F*(2,57) = 12.56, *p* < 0.01, η^2^ = 0.31). Univariate analyses conducted for picture and odor conditions separately indicated that the effect of delay was significant for the odors (*F*(2,57) = 15.34, *p <* 0.01, η^2^ = 0.35). Tukey post hocs for multiple comparisons indicated significant differences between the 15-min and 48-h condition (*p <* 0.01) and between the 15-min and 1-week (*p <* 0.01), but the difference between 48 h and 1-week (*p* = 0.15) was not significant. Thus, the pattern of memory performance was that hit rates decreased as a function of delay for odors but not for pictures.

**Figure 12:**
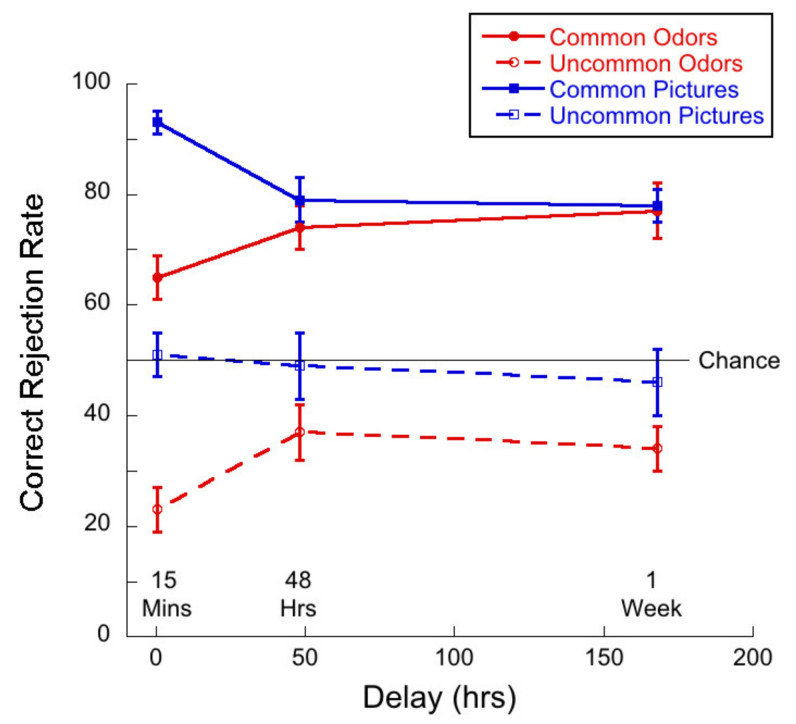
Correct rejection rate as a function of delay for each of the four test conditions. Given that this was a “monadic” task, guessing would result in a hit rate of 50%.

Figure 12 illustrates proportion of correct rejections as a function of delay for common and uncommon pictures and odors.

A 2 (commonness) × 3 (delay) mixed ANOVA on correct rejections for the picture stimuli revealed no significant interaction (*F*(2,57) = 1.25, *p* = 0.30, η^2^ = 0.04) between commonness and delay. There was a main effect of commonness (*F*(1,57) = 111.76, *p* < 0.01, η^2^ = 0.66) and there was a trend towards an effect of delay (*F*(2,57) = 2.59, *p* = 0.08, η^2^ = 0.08). Thus, the CR rate was higher for common than for uncommon pictures and there was a trend towards a decrease in correction rate with delay for both conditions.

A 2 (commonness) × 3 (delay) mixed ANOVA on correct rejections for the odor stimuli revealed no significant interaction (*F*(2,57) = 0.42; *p* = 0.66, η^2^ = 0.01) between commonness and delay. There was a main effect of commonness (*F*(1,57) = 199.43, *p* < 0.01, η^2^ = 0.78). The effect of delay (*F*(2,57) = 3.15, *p* = 0.05, η^2^ = 0.10) narrowly missed significance. Thus, CR rate was higher for common than for uncommon odors and there was a trend towards performance increasing with delay for both odor conditions.

We conducted a 2 (stimulus type) × 3 (delay) mixed ANOVA of the correct rejections for common pictures and odors. We found a significant interaction between variables (*F*(2,57) = 9.28, *p* < 0.01 η^2^ = 0.25). There was a main effect of stimulus type (*F*(1,57) = 18.57, *p* < 0.01, η^2^ = 0.25) as memory was better for pictures than for odors, but no main effect of delay (*F*(2,57) = 0.26, *p* = 0.77, η^2^ = 0.01). Univariate analyses conducted for picture and odor conditions separately indicated that the effect of delay was significant for the pictures only (*F*(2,57) = 8.99, *p <* 0.01, η^2^ = 0.24). Tukey post hocs for multiple comparisons indicated significant differences between the 15-min and 48-h condition (*p <* 0.01) and between the 15-min and 1-week (*p <* 0.01), but not for the difference between 48 h and 1-week (*p* = 0.97). Thus, the pattern of memory performance was that CR rates decreased as a function of delay for pictures but not for odors.

**Figure 13:**
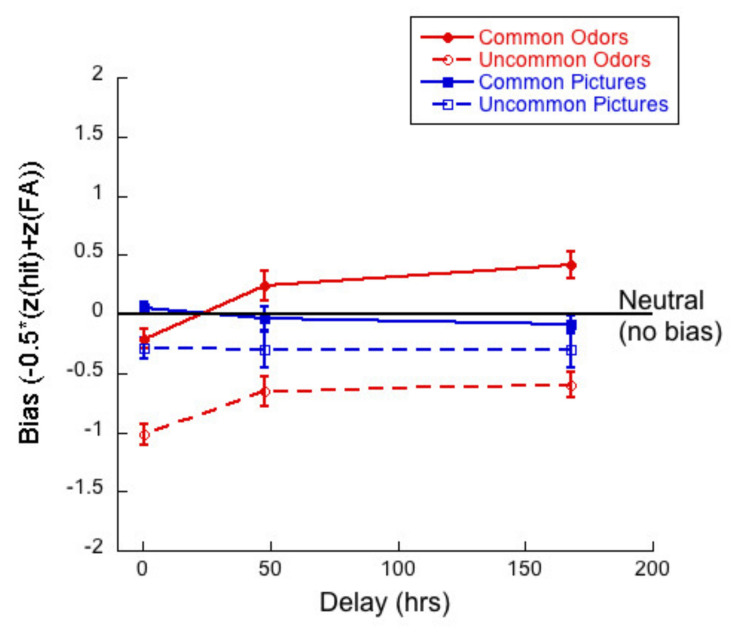
Bias as a function of delay for each of the four test conditions. Negative values reflect a “liberal” criterion (greater willingness to say “yes”).

Figure 13 illustrates bias/criterion as a function of delay for common and uncommon pictures and odors.

A 2 (commonness) × 3 (delay) mixed ANOVA on bias for the picture stimuli revealed no significant interaction (*F*(2,57) = 0.18; *p* = 0.84, η^2^ = 0.01) between commonness and delay. There was a main effect of commonness (*F*(1,57) = 9.67, *p* < 0.01, η^2^ = 0.15), and no main effect of delay (*F*(2,57) = 0.26, *p* = 0.77, η^2^ = 0.01). Thus, bias was closer to “neutral” for common pictures and more “liberal” for uncommon pictures. Bias did not change with delay.

A 2 (commonness) × 3 (delay) mixed ANOVA on bias for the odor stimuli revealed no significant interaction (*F*(2,57) = 0.59; *p* = 0.56, η^2^ = 0.02) between commonness and delay. There was a main effect of commonness (*F*(1,57) = 131.51, *p* < 0.01, η^2^ = 0.70) and of delay (*F*(2,57) = 11.48, *p* < 0.01, η^2^ = 0.29). Thus, bias was significantly different for common odors (closer to “neutral”) than uncommon odors (more “liberal”) and bias increased with delay (becoming more “conservative”) with delay for common odors and more “neutral” with delay for uncommon odors.

## 6. Discussion

In this paper we addressed the question: Does olfactory memory rely on different mechanisms than visual memory? In particular, we asked whether “novelty detection” is an important mechanism in long-term olfactory memory. We hypothesized and found that visual and olfactory memory depend differentially on ‘novelty’ detection as demonstrated with differential hit and correct rejection rates and patterns of responses with delay in the monadic “yes/no” task. We also expected that olfactory memory would suffer less forgetting than visual memory. Specifically, we expected to find main effects of delay for pictures but not for odors. Our data did not support that hypothesis. Finally, we hypothesized that memory of uncommon stimuli would decay similarly to memory of common stimuli, as demonstrated with no interactions between the variables of delay and “commonness”. This was only partially supported as there were some significant interactions, particularly in the case of d’.

### 6.1. Forgetting Curve(s) Not Flat for Odors

The results from this experiment demonstrate that memory (as measured by percent correct in a forced-choice task (with option of responding “neither”) or as sensitivity in a “monadic” task) decreased with delay for both odors and pictures. We do not find evidence for flat forgetting curves in olfaction as reported previously [16,18,23], and for a review see [22], although forgetting curves in the forced-choice tasks were flatter than they were when odor memory was measured by means of a monadic task.

The lack of a flat forgetting curve for common odors is in accord with results of Cornell Kärnekull and colleagues [10]. In a monadic task, those authors found that the forgetting curves for visual and olfactory stimuli were not fundamentally different and that the forgetting curve for olfactory stimuli was not flat. That study differs from the one described here in a number of ways. Most importantly, learning was intentional and the authors instructed participants to try to identify odors in the learning phase. Odors that were easily identified at learning and at test produced high hit rates (97%). It is not possible to disentangle whether participants were remembering the odors or the names of identified odors. However, the fact that hit rates dropped to 55% after four days for odors that were not identified consistently, which is consistent with the present findings for common odors, suggests that memory for consistently identified odors were not reflecting memory for the odor per se.

As discussed below (and previously in the paper), the main reason for the flat forgetting curves often observed in odor memory studies might be an artifact of using forced-choice methods to quantify memory or forgetting.

### 6.2. Forced-Choice Testing of Odor Memory

Some studies of odor memory employ forced-choice methods. There is a fundamental problem with forced-choice methods in memory studies, as described in the Introduction, which is that a correct answer can be arrived at in one of two ways. It can be the result of comparing the test stimuli with an internal representation of the target (i.e., a memory trace). Template-matching may be the primary mechanism in visual memory and is the standard interpretation of a correct response. On the other hand, a correct response can also be arrived at with no recollection of the target, if the participant can identify the foil as a novel stimulus. If the foil is detected as novel, the participant could deduce that the other stimulus, which the participant cannot match to any internal representation, is actually the target. Thus, template-matching may not be the only mechanism involved in making a correct response.

Confounding two possible memory mechanisms is not the only problem with forced choice methods in the context of memory. In an odor memory experiment using 2AFC methods, participants can still feel that they have not smelled either of the odors before. That is, both odors in the test could seem to the participant to be novel. The appropriate response in such a situation is to respond that neither of the odors have been smelled before. We, therefore, added the option of responding ‘neither’ in our forced-choice task and found that participants chose ‘neither’ in about 30% of all trials for common odors in both the incidental and intentional learning conditions. This suggests that in a typical 2AFC memory task with common odors, on about 30% of trials participants would be of the opinion that neither of the two odors presented had been smelled before. However, since an answer is required, participants would have to guess because both stimuli are evaluated to have about the same degree of novelty. This means that percent correct may be inflated by about 15% in a standard paradigm. The problems with using 2AFC methods are not unique to studies of memory for odors.

Overall memory for odors in the current forced-choice task was apparently low (55–60% correct), but since the task included “neither” as a response option, chance was 33.33%. Even considering that performance could have been ~15% (about half of “neither” responses) higher had this been a typical 2AFC task, performance was lower than the ~85% correct performance levels reported by Lawless [17] for delays comparable to ours. Part of this difference might be a result of the odors used, as we know from our data (see Supplementary Figure S1) that memory for some odors is much better than others.

The most serious problem with forced-choice methods in memory studies is that they confound two different memory mechanisms: matching a test stimulus to a memory trace vs. novelty detection. This problem is avoided when using monadic methods as discussed earlier in the paper.

### 6.3. Monadic Testing Should Be Used in Memory Experiments

In monadic testing, one stimulus at a time is presented in the test phase and the participant is required to decide whether the stimulus was encountered in the learning phase. For the intentional learning condition, Figure 4 shows that there was no significant drop in d’ over delay for common odors, whereas d’ for common pictures decayed significantly with delay. Figure 5 and Figure 6 show a decreasing hit rate and a constant correct rejection rate for common odors. For pictures, hit rate stayed constant over time, whereas correct rejection rate decreased with delay. The significant drop in hit rates for common odors could have been caused by a more conservative response criterion developing over delays. Even though response bias is close to zero for all delays, it does increase slightly with delay, as shown in Figure 7. If, however, a more conservative response criterion is the reason for the significant drop in hit rates, we would expect the correct rejection to increase. Figure 6 shows that this is not the case. From Figure 7 we also see that bias for common pictures stayed constant at a value around zero. These data, thus, support the hypothesis that memory for common pictures and common odors depend differently on target recognition and ‘novelty’ detection.

### 6.4. Incidental vs. Intentional Learning

For both modalities, in intentional conditions, where it is revealed to participants that they will be tested on their memory for the stimuli with which they are presented in the learning phase, verbalization and imagery might help participants to encode stimuli into memory. Comparing results from the intentional learning condition, as depicted in Figure 4, Figure 5, Figure 6 and Figure 8, with Figure 10, Figure 11, Figure 12 and Figure 13 from the incidental learning condition, we see that the pattern of results in the two learning conditions was very similar. Sensitivity (d’), hit and correct rejection rates changed with delay in the same manner in the two conditions. Thus, also for incidental learning, memory for common pictures and common odors depended differently on target recognition and ‘novelty’ detection. Monadic testing thus allows for more detailed study of underlying mechanisms. Results of the forced choice task, for all four types of stimuli, show the same pattern for the incidental and intentional condition (see Figure 2 and Figure 8). That is, in our case, intentional learning did not significantly influence memory for common or uncommon odors and pictures. This strongly suggests that for the varied olfactory and visual stimuli we used, focused encoding strategies in the intentional condition, based, for example, on verbalization or imagery, did not influence results, but rather memory in all cases relied on the perceptual content of the stimuli. Our results in memory for odors are in agreement with previous studies that found no differences between intentional and incidental learning conditions in a 2AFC experiment [16] and in monadic tasks [53,54], and with Zucco [55], who did not find any evidence of verbal encoding strategies.

### 6.5. Uncommon vs. Common Stimuli

Comparing percent correct in the forced-choice task (Figure 2 and Figure 8) and d’ in the monadic measurements (Figure 4 and Figure 10), we found that uncommon stimuli in both modalities were remembered less well than common stimuli. For visual stimuli this is in accordance with previous investigations [10,17] and supports the hypothesis that visual memory is largely based on identification. Is this also the case for olfactory stimuli, as suggested by Cornell Kärnekull and colleagues [10]?

The plots of hit rates (Figure 5 and Figure 11) show that these were around 80% for uncommon odors, with very little decay with delay. Hit rates for common odors decreased from ~75% to ~50% (chance) after one week. If odor memory were based on identification or other types of semantic knowledge, we would expect hit rates to be higher for common than for uncommon odors, which was not the case in our data. Correct rejections, on the other hand, were significantly higher for common odors than for uncommon odors (Figure 6 and Figure 12). Our data, therefore, do not support the hypothesis that odor memory is largely based on identification. Bias for uncommon odors (Figure 7 and Figure 13) was negative, corresponding to a very liberal response criterion. Participants responded “yes” much more often than they responded “no”, when tested with uncommon odors. These results suggest that uncommon odors are treated as a separate category of ‘strange odors’. When an uncommon odor is smelled in the test phase, participants scored around 80% hits and around 70% false alarms and both were roughly constant over delay. The small difference in hit and false alarm rates produce the small d’ values for uncommon odors. In line with the suggestion that uncommon odors are treated in memory as a category, with only small variations between members of the category, we find that the number of incorrect responses that were “neither” (Figure 3 and Figure 9) in the forced-choice task was smaller for uncommon odors than for common odors, meaning that in most cases when uncommon odors were smelled in the test phase, they were rarely both rejected

Both hit rates and correct rejection rates for common pictures were larger than hit rates and correct rejection rates for uncommon pictures. Contrary to this, hit rates were lower for common odors than for uncommon odors, whereas correct rejection rates were higher for common odors than for uncommon odors (Figure 5, Figure 6, Figure 11 and Figure 12). These results suggest that long term odor and visual memory are governed by different mechanisms.

### 6.6. Olfactory Memory vs. Odor Memory and Ecological Validity of Measurements

In agreement with previous studies of odor memory [16,18,19,56] we found that percent correct (in forced-choice tasks) were not larger than 55%, which, even if corrected to be comparable with other studies, would not be larger than 70%. That is, percent correct for odor memory is always well below 100% as opposed to the case of visual memory, which was nearly perfect at short delays. Similarly, d’ (in monadic tasks) was never larger than 1.25. What utility is a memory system with this low accuracy?

Reliable short term or working memories are crucial for speech comprehension and for reading. Long term visual memory of spatial layout is necessary for wayfinding as well as for object and face recognition, without which most of social and societal activity would not be possible. There are, thus, good behavioral reasons for accurate, high capacity visual and auditory memories.

Measuring odor memory by presenting odors in a bottle to participants treats odors as if they were objects to be perceived on their own. Low percent correct and small d’ in long term odor memory tests suggest that odors are not there to be remembered as ‘separate entities’ or ‘odor objects’. Except for a few professionals, such as cooks and perfumers, being able to remember odors per se, does not seem to be an ecologically important capability. On the other hand, there is no doubt that olfactory memory, referring to memories relying on olfactory input, is ecologically important. Examples include autobiographical memories induced by odors, context dependent memories and olfactory memory to help us feel at home and to detect when something is out of place [22,47]. These memories are characterized by their relationships with events and environments, as opposed to being memories of the odor itself.

### 6.7. Is Odor Memory ‘Special’?

Even though the present study treated odors as separate entities, we believe the results contain hints about differences between visual and olfactory memory. We found different rates of decay of hit and correct rejection rates for common pictures and common odors and different hit and correct rejection rates for common and uncommon stimuli. These results suggest that long term odor memory is strongly dependent on ‘novelty detection’ and less on a memory trace of odors encountered in the learning phase. This is very different from the case of memory of pictures where participants have a robust internal representation of pictures.

We agree with Engen and Ross [16] and others [18,19,56] that olfaction is ‘special’, but not because of a flat forgetting curve, which we do not find. Rather, what seems to make long term odor memory special in our data is that it relies more on ‘novelty detection’ than on recognizing a previously smelled target. The low accuracy of odor memory also distinguishes it from visual memory. This corresponds to what has been found for odor and picture identification [40,45,46,57,58,59], namely that it is much more difficult to name odors than pictures, especially when decontextualized. Visual identification is important because it allows preparation for appropriate response (fleeing, stepping aside, embracing, etc.) In olfaction we have no time for such preparation, since the potentially harmful substance is already in the body when it is perceived. Being able to detect a novel, and therefore, potentially dangerous, substance is important in olfaction in order to generate the appropriate behavior of holding one’s breath and moving away. The results of the present experiments suggest an important role of novelty detection in long term odor memory and provide further support for the theory suggested by Köster and his colleagues [47].

### 6.8. Limitation

As with any study, ours has some limitations. First, although we tested all available participants at our college location, some of our conditions had more participants than others. On the other hand, standard errors were quite low and the results of the two experiments were so similar that it seems that our results were quite robust. Second, it is true that baseline levels of performance were not the same for all conditions. We believe that this is a function of differences between memory for odors and pictures (people remember pictures better than odors). It is possible that a different set of stimuli could have generated more similar baseline levels of performance, but we do not believe that would explain away the differences we observed between memory for odors and pictures.

## Data Availability

Data are available upon request to the authors.

## Supplementary Materials

The following are available online at https://www.mdpi.com/article/10.3390/brainsci11091146/s1, Figure S1: odor-by-odor analysis from incidental learning condition.

## Appendix A

**Table A1:** Common and uncommon odors, in pairs.

Target	Quantity	Foil	Quantity
Lemon	2 mL	Lime	00.5 mL
Vanilla Extract	1 mL	Almond Extract	00.25 mL
Paprika	0.075 g	Curry	0.25 g
Oregano	0.6 g	Thyme	0.2 g
Clove	0.04 g	Cinnamon	0.25 g
Peanut	10.5 g (4 halves)	Shoe Polish	0.035 g
Soy Sauce	0.5 mL	Cat Food	10.1 g (4 pieces)
Honey	6 g	Ginger	0.2 g
Rose	1 mL	Ivory Soap	10.2 g
Chocolate	2 g (4 chips)	Coffee	0.6 g (4 beans)
1,1-dimethyl-2-phenylethanol	0.5 mL	1-phenylethyl acetate	0.5 mL
2-Phenyleth. Eth ether	0.5 mL	4-Phenylbutan-2-One	0.5 mL
2,6-dimethylhept-5-enal	0.5 mL	Methyl Benzoate	0.5 mL
5-meth. Haptan-3-one oxime	0.5 mL	Plicatone	0.5 mL
Citrowanil	0.5 mL	3, 7-demethyloctanenitrile	0.5 mL
Diethyl Malonate	0.5 mL	3, 4, 5, 6, 6-pentameth. Hept-3-en-2-one	0.5 mL
Linayl Acetate	0.5 mL	Nonyl Acetate	0.5 mL
Octan-1-ol	0.5 mL	2-phenyleth penth ether	0.5 mL
Terpinyl Acetate	0.5 mL	Cashmeran	0.5 mL
Vigoflor	0.5 mL	Verdox	0.5 mL
