# Supplementary material for: Is Novelty Detection Important in Long-Term Odor Memory?
Source: Brain Sci. 2021 Aug 29;11(9):1146. doi: 10.3390/brainsci11091146 (PMC8470488; doi:10.3390/brainsci11091146)
Supplement: Supplementary file 1 [file brainsci-11-01146-s001.zip › brainsci-1303842-supplementary.pdf]

Results: Odor-by-Odor Analysis

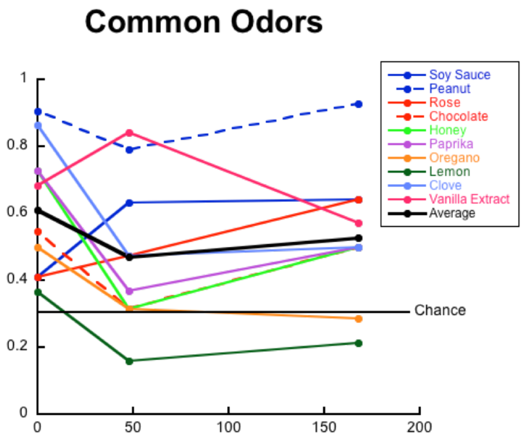

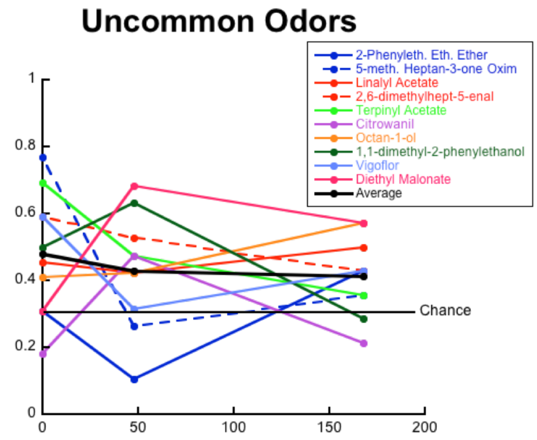

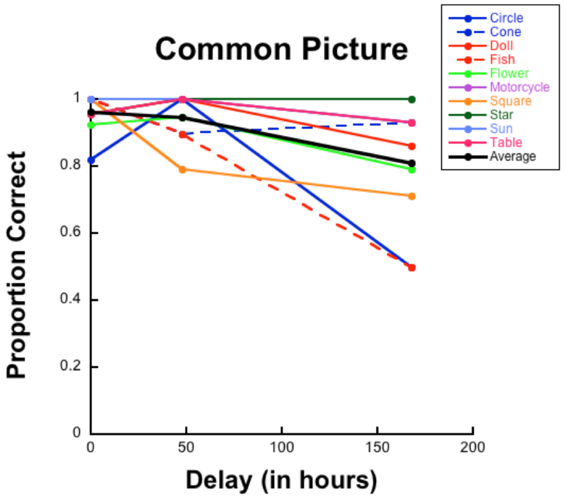

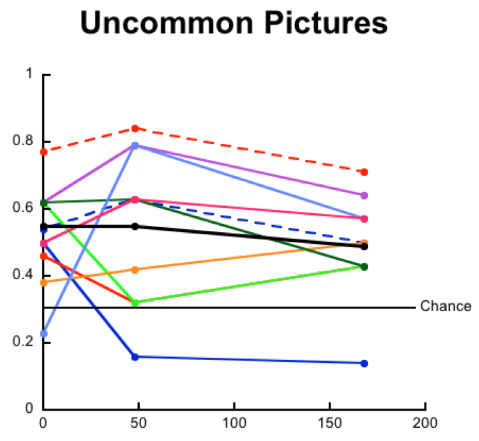

Recognition memory depends upon the particular stimulus to be remembered.

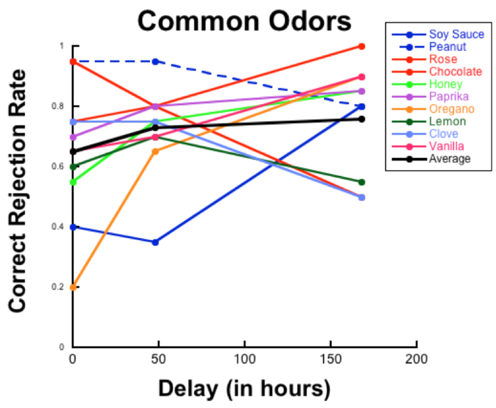

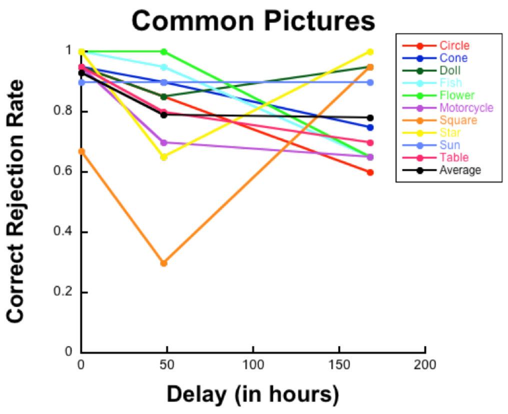

Correct Rejection Rate (which cannot be identified in forced-choice tasks) vary with delay and depend upon the particular stimulus to be remembered. Correct rejection rate increases for many odors but decreases for most pictures.
Note: Correct Rejection Rate reflects "rejecting" the odor that was paired with a target odor in the 3AFC task.

Source: Cameron, E.L. Houzenga, C., Köster, E.P. & Møller, P. (2015, April). *Olfactory and visual memory: Same or different?* Poster presented at the Annual Meeting of the Association for Chemoreception Sciences, Bonita Springs, FL.
